# Biotransformation of Waste Bile Acids: A New Possible Sustainable Approach to Anti-Fungal Molecules for Crop Plant Bioprotection?

**DOI:** 10.3390/ijms23084152

**Published:** 2022-04-08

**Authors:** Alessandro Grandini, Daniela Summa, Stefania Costa, Raissa Buzzi, Elena Tamburini, Gianni Sacchetti, Alessandra Guerrini

**Affiliations:** 1 Department of Life Sciences and Biotechnology, University of Ferrara, Via L. Borsari 46, 44121 Ferrara, Italy; grnlsn1@unife.it (A.G.); bzzrss@unife.it (R.B.); gianni.sacchetti@unife.it (G.S.); alessandra.guerrini@unife.it (A.G.); 2 Department of Humanities, University of Ferrara, Via Paradiso 12, 44121 Ferrara, Italy; daniela.summa@unife.it; 3 Department of Chemical, Pharmaceutical and Agricultural Sciences—DOCPAS, University of Ferrara, Via L. Borsari 46, 44121 Ferrara, Italy; 4 Department of Environmental and Prevention Sciences, University of Ferrara, Via L. Borsari 46, 44121 Ferrara, Italy; tme@unife.it

**Keywords:** biotransformation, waste bile acids, secosteroids, in vitro antifungal activity, phytopatogens

## Abstract

Phytopathogenic fungi are among the main causes of productivity losses in agriculture. To date, synthetic chemical pesticides, such as hydroxyanilides, anilinopyrimidines and azole derivatives, represent the main treatment tools for crop plant defence. However, the large and uncontrolled use of these substances has evidenced several side effects, namely the resistance to treatments, environmental damage and human health risks. The general trend is to replace chemicals with natural molecules in order to reduce these side effects. Moreover, the valorisation of agri-food industry by-products through biotransformation processes represents a sustainable alternative to chemical synthesis in several sectors. This research is aimed at comparing the anti-phytopathogenic activity of waste bovine and porcine bile with secosteroids obtained by biotransformation of bile acids with *Rhodococcus* strains. The ultimate goal is to apply these natural products on food crops affected by phytopathogenic fungi.

## 1. Introduction

Fungal phytopathogens represent one of the main causes of production depletion in agriculture [1,2].

The only tool available nowadays to fight against the plants attacked by fungi is represented by synthetic pesticides. The wide use of synthetic compounds in agriculture has certainly made it possible to maintain, if not increase, agricultural production in the last century, in the face of a continuous increase in demand for agricultural products [3]. However, their use has led to the emergence of numerous problems, such as soil and water pollution and drug resistance in phytopathogens, making the management of crop protection treatments ever more difficult, with effects on the environment that are even more important [4]. The first compound recognised for its fungicidal activity was discovered by Bénédict Prévost in 1807, through the ascertaining of *Tilletia* spore germination inhibition by metallic copper in soil [5]. Otherwise, the first organic fungicide, an organomercurial compound, was synthesised at the beginning of the 20th century. Later on, several studies have been carried out on the production of many synthetic fungicides, such as 2-methoxyethyl silicate and 2-hydroxyphenyl mercury, effective against some fungi such as *Fusarium* spp. and *Dreschlera* spp. [6]. There are many examples of antifungal agents in the literature and on the market, including benzimidazoles, dithiocarbamates, strobilurins [7] and azole molecules such as triazoles, which are widely used in agriculture [8]. In the face of such a varied availability of effective compounds, their common characteristics of great stability in the environment are evident, determining important accumulation phenomena in the environment (long degradation times) that affect the agricultural products themselves and the health of consumers. In fact, since the 1950s and 1960s, several studies have been evidencing how biocides used in agriculture can affect human health in the long term [9], by dermal contact, inhalation or ingestion of contaminated food or water [10], provoking adverse health effects at endocrine, immunological and neurological levels, as well as carcinogenic risk and premature births [9].

Among the environmental risks of large and uncontrolled use of fungicides and synthetic agripharmaceutical products, there are toxicological effects on not-targeted species, as earthworms or soil microorganisms, leading to dramatic changes in the overall ecological balance [11,12,13,14].

Given the above premises, a growing need is by the use of effective natural—rather than synthetic-compounds. In fact, many scientists are focusing on biopesticides and natural compounds for a more sustainable management of crop protection strategies [13,15]. Substances with antifungal activity can be produced by microorganisms, plants, or animals, and themselves can be used as antagonists for a biocompatible and more sustainable defence of crops [16]. Moreover, plant extracts (e.g., essential oils) have been demonstrated to inhibit the production of mycotoxins from many fungal species [17,18], evidencing their effective role also as tools to prevent post-harvest infections [19]. However, the natural compounds for crop defence share the following advantages: reduced toxicity towards organisms that do not represent the target of action (high selectivity); reduced persistence in the environment; low or no toxicity to mammals (absent in many cases); lower risks for operators linked to their use; and lower risk of developing resistance [20]. The disadvantages compared to synthetic compounds can instead be summarised in a more reduced effectiveness, also due to a reduced stability in the environment [21]. On the other hand, the tendency to seek ever more sustainable solutions to crop management becomes a need that also derives from the greater attention of consumers, who show more and more preferences towards products with an allegedly low residue of pesticides and with reduced environmental impact [22].

In addition, new currents of thought relating to defence tend to highlight how the implementation of sustainable plant protection is increasingly aimed at putting into practice mechanisms aimed not at eliminating the pathogen, but rather at containing it. In fact, every organism, even if pathogenic, performs a function within the ecosystem for which its eradication from the environment would cause imbalances between existing populations [23,24,25,26].

Natural active substances with antifungal properties currently used in agriculture are mainly phytoalexins and phytoanticipins [27]. There are many examples in literature of effective crop control by employing natural compounds or extracts (e.g., plant extracts) in pest control, even if most of them reflect in vitro evidence. A coumarin isolated from *Citrus paradis* exhibits antifungal activity both in vitro and in vivo against *Penicillium italicum* and *Penicillium digitatum* [28]; chlorogenic acid, pyrogallol, pyrocatechol, phenol and resorcinol can inhibit *Botryodiplodia Theobroma* that causes Java black rot (one of the most significant postharvest diseases of the sweet potato); sesquiterpenes exhibit antifungal activity against *R. solani* [29], etc. [25].

An alternative approach to the use of natural compounds or extracts from natural sources can be the production of new antifungal products by fermentation or biotransformation technologies, starting from low-value substrates or from by-products of agri-food processes [30]. These methodologies are commonly used to improve existing technologies and to enhance low-value molecules and thus could be profitably used in this area as well. Bile acids are surfactant steroid compounds found in the digestive tracts of vertebrates [31]. Their main physiological function is to emulsify bile lipids and dietary lipids. Bile acids may be easily recovered as side products from the food industry and thus represent a discard product, from cattle or pig slaughterhouses [32], that is used to produce bile acid substitutes for humans [33]; surface active molecules are also known to express antifungal activity, thus we thought that it could be an interesting approach to use modified bile acids as antifungal agents [34,35].

Chemical synthesis involves a number of disadvantages due to the use and disposal of solvents and high temperatures with consequent risks to operators. To overcome the problems arising from chemical synthesis it is possible to exploit the microbial biotransformation approach [36] as it has a number of advantages over chemical synthesis, such as regio- and stereospecific functionalisation of molecules, the possibility to perform complex reactions in one-pot one-step and finally greater environmental sustainability (mild reaction conditions and aqueous media) [37].

Thus, this study is focused on the evaluation of the anti-phytopathogenic activity of bile acids and 9,10-secosteroids, a newly-biosynthesised compound derived from biotransformation of bile acids, hypothesizing that because they are new molecules that have never been used for this purpose, they can bypass the issues with traditional pesticides. As a potential target, ten phytopathogenic fungi that cause multiple problems affecting the agricultural sector worldwide have been identified.

## 2. Results

In this study ten phytopathogenic fungi, causing multiple damage to many important crops every year, were chosen.

Bovine and porcine bile acids and the corresponding secosteroids have been evaluated, together with the biotransformation products, for their inhibition of fungi growth.

Table 1 shows the phytopathogenic fungi used for this study, the target plant crops and the phytopathyes resulting from the infection.

### 2.1. Bile Acids and Secosteroids Deriving from Biotransformation

**Figure 1:**
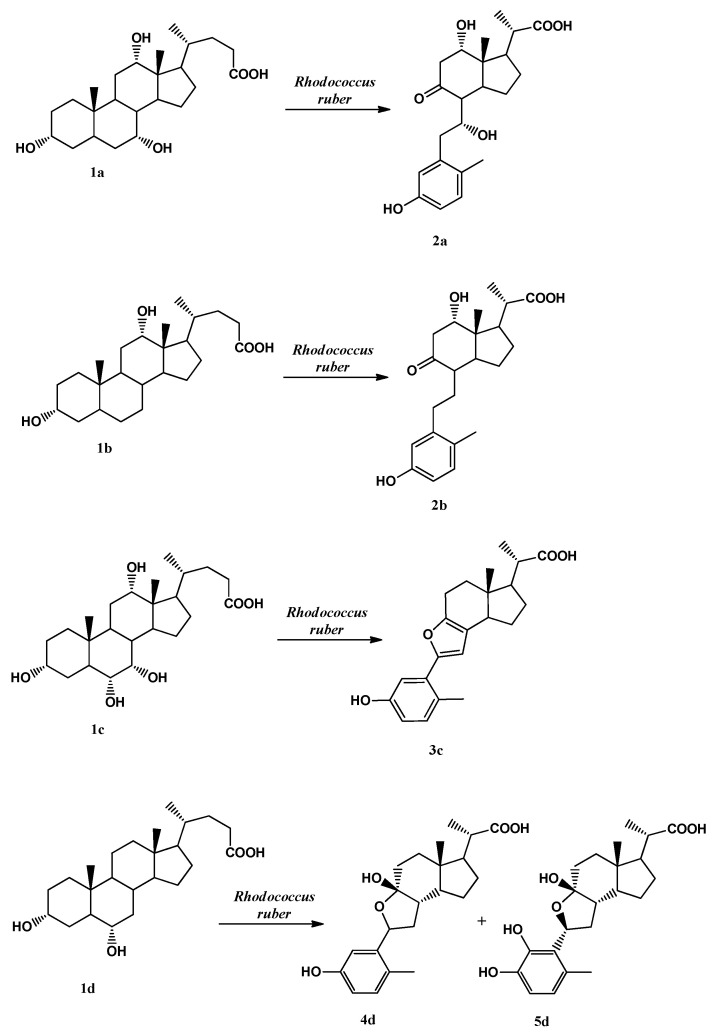
Bile acids and biotransformation products.

In Figure 1, the structures of bile acids (used as substrates) and of secosteroid derivatives (obtained after biotransformation of bile acid with *Rhodococcus rhodnii*) are reported.

Through the biotransformations of cholic acid **1a**, deoxycholic acid **1b** and hyocholic acid **1c**, only one secosteroid product is obtained (**2a**, **2b** and **3c**, respectively), while in the biotransformation carried out with hyodeoxycholic acid **1d** in the extract, the presence of two biotransformation molecules, characterised as secosteroids, was evidenced (**4d** and **5d**). The **5d** product derives from the hydroxylation of the aromatic ring formed after the opening of the B ring of the steroid nucleus. Bile acids deriving from porcine and bovine bile and the respective products obtained through biotransformation with strains belonging to the *Rhodococcus* genus extracts employed to be tested as potential antifungal agents against phytopathogens microorganisms have been obtained and described in Costa et al. [70,71].

### 2.2. Preliminary Screening for Antifungal Activity

**Figure 2:**
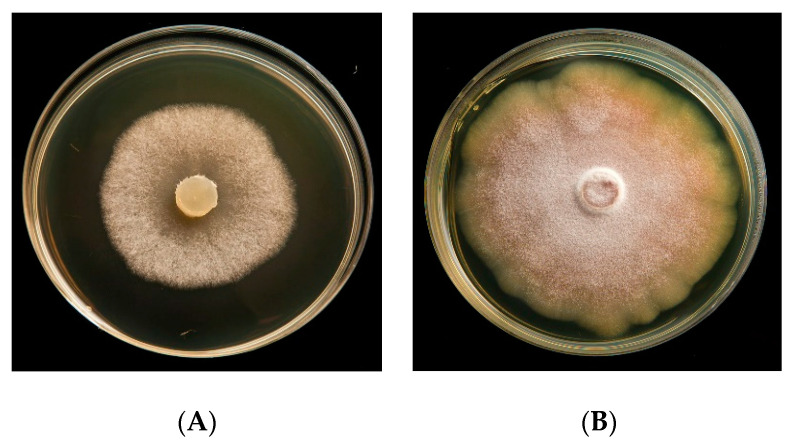
Grow inhibition test (**A**), negative control DMSO (**B**) on *Phoma betae*.

In this study, a preliminary screening of antifungal activities has been conducted. After appropriate incubation time, the inhibition percentage of diametrical growth was observed and compared to the negative control (DMSO) as showed in Figure 2.

Table 2 and Table 3 show the antifungal activity results obtained respectively with bile acids (**1a,d**) and with secosteroids (**2a,b**, **3c** and **4d + 5d**) expressed as growth inhibition percentage related to the negative control.

Results of the screening using bile acids at the concentration of 1000 μg/mL showed that *P. betae*, *S. minor* and *F. moniliforme* were sensitive to the tested compounds (with a growth inhibition equal or greater than 80%). Namely, *P. betae* proved to be particularly sensitive to three of the bile acids tested: deoxycholic acid **1b**, hyocholic acid **1c** and hyodeoxycholic acid **1d**, with an inhibition percentage of 100%, 86% and 87%, respectively. Otherwise, *S. minor* and *F. moniliforme* underwent an evident growth inhibition only in case of the use of deoxycholic acid **1b** with growth inhibition of 83 and 85%, respectively.

Significant inhibition of fungal growth has been obtained using secosteroids. In addition to *F. moniliforme* and *P. betae*, which were, respectively, sensitive to the **4d + 5d** mixture with 95% inhibition and to the **3c** extract with 100% inhibition, other fungi have undergone growth reduction with some secosteroids. For example, *P. ultimum* evidenced growth inhibition with three different secosteroid extracts, i.e., 85% inhibition using the **2a** extract, 87% using **3c** and 100% with the extract containing the **4d + 5d** blend.

*S. minor* was also found to be particularly sensitive to some secosteroid extracts, obtaining inhibition yields from 81% with extract **2a** up to yields of 93% with extract **3c**.

Finally, *S. sclerotiorum* was found to be particularly sensitive to the samples containing the secosteroids **3c** and **4d + 5d** with growth inhibition yields of 100% and 98%, respectively.

### 2.3. Inhibition of Diametrical Growth Test

Fungi that showed a growth inhibition equal to or more than 80% compared to controls were further analysed by repeating the test with a concentration range between 1000 and 25 µg/mL. The IC_50_ values evidenced by bile acids (**1a,d**) and secosteroids (**2a,b**, **3c** and **4d + 5d**) are reported in Table 4 and Table 5, respectively. Fluconazole was used as positive control at two different concentrations (50 and 100 µg/mL). The computation of the IC_50_ was carried out using the GraphPad Prism software.

Bile acids and secosteroids were particularly active against the pathogens *F. moniliforme*, *P. betae*, *P. ultimum*, *S. minor* and *S. sclerotiorum*. While cholic acid did not show a particular anti-phytopathogenic activity, the extract containing secosteroid **2a** proved to be active against *P. ultimum* and *Sclerotinia minor*, with IC_50_ of 677.0 and 730 μg/mL, respectively. Deoxycholic acid **1b**, has been shown to be effective against various fungi, in particular against *P. betae* (IC_50_ of 22.14 μg/mL). The extract containing the secosteroid **2b** showed activity against *S. minor* and *S. sclerotiorum*: the test against *S. minor* showed an IC_50_ lower than that of the corresponding pure bile acid, while against *S. sclerotiorum* IC_50_ it was 133.50 μg/mL.

The results obtained with hyocholic acid **1c** and the corresponding secosteroid **3c** showed that both the biotransformed compounds were effective against *P. betae*, with an IC_50_ of the **3c** compound about ten times lower than the starting bile acid. As for *S. minor*, only product **3c** shows a significant growth inhibition activity with an IC_50_ value of 67.90 μg/mL.

*S. sclerotiorum* is sensitive to both **1c** and **3c** but with highly different IC_50_ values: in the first case the value was 712.80 μg/mL, while in the case of the extract containing the secosteroid **3c** the IC_50_ is equal to 18.49 μg/mL (value almost forty times lower than **1c** bile acid).

Finally, hyodeoxycholic acid **1d** showed activity only against *P. betae* and *P. ultimum* with IC_50_ values of 196.6 and 284.1 μg/mL, respectively.

The biotransformation mixture, containing the secosteroids **4d + 5d** instead, showed activity against *F. moniliforme* and *S.sclerotiorum* (unlike the starting bile acid for the biotransformation), while against *P. ultimum* the secosteroids **4d + 5d** show IC_50_ significantly lower than that of hyodeoxycholic acid (176.2 μg/mL).

**Figure 3:**
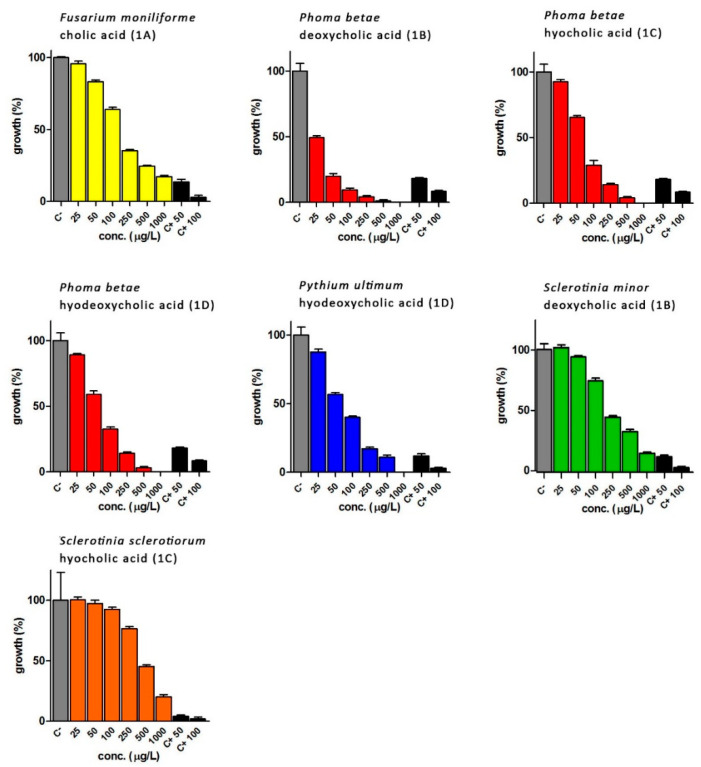
Growth inhibition of phytopathogenic fungi treated with bile acids (**1a,d**). FM = *Fusarium moniliforme*; PB = *Phoma betae*; PU = *Pythium ultimum*; SM = *Sclerotinia minor*; SS = *Sclerotinia sclerotiorum*. C− = DMSO; C+ 50 = fluconazole 50 μg/mL; C+ 100 = fluconazole 100 μg/mL. **1a** = cholic acid, **1b** = deoxycholic acid, **1c** = hyocholic acid, **1d** = hyodeoxycholic acid.

**Figure 4:**
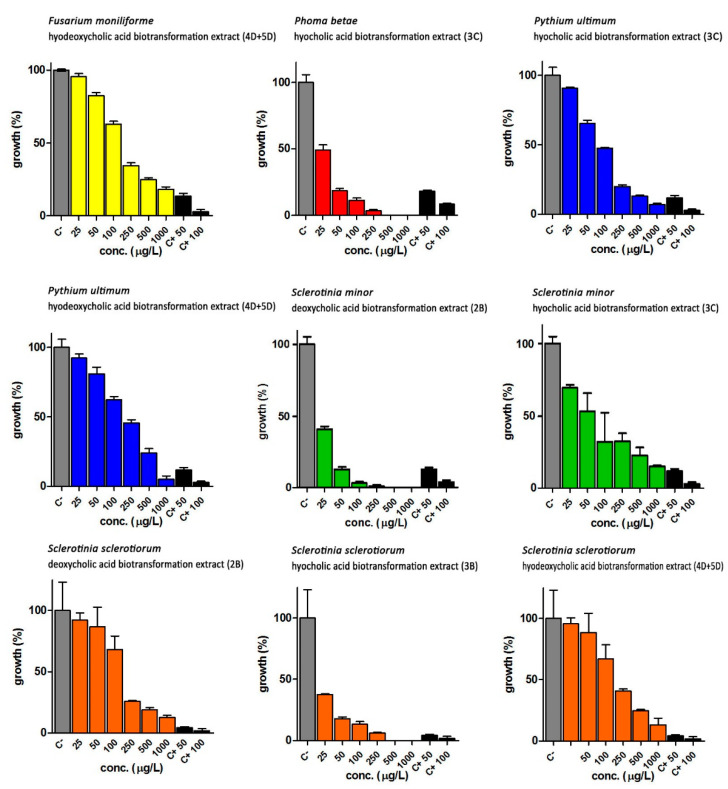
Growth inhibition of phytopathogenic fungi with secosteroids (**2a**,**b**, **3c** and **4d + 5d**). FM = *Fusarium moniliforme*; PB = *Phoma betae*; PU = *Pythium ultimum*; SM = *Sclerotinia minor*; SS = *Sclerotinia sclerotiorum*. C− = DMSO; C+ 50 = fluconazole 50 μg/mL; C+ 100 = fluconazole 100 μg/mL. **2a** = secosteroid from cholic acid, **2b** = secosteroid from deoxycholic acid, **3c** = secosteroid from hyocholic acid, **4d + 5d** = secosteroids from hyodeoxycholic acid.

In Figure 3 and Figure 4, the results obtained using bile acids and biotransformation extracts at different concentrations (range 25–1000 µg/L) as potential anti-phytopathogenic agents are reported.

In Figure 3, the anti-phytopathogenic activity of bile acids is shown. In most cases the fungal strains treated with the synthetic antifungal compound fluconazole evidenced the highest sensitivity. However, only in the case of *Phoma betae*, treated with deoxycholic acid (**1b**), the results were comparable to those obtained with fluconazole at 50 and 100 μg/mL.

As in the previous case, analysing the activity of secosteroids compared to that of fluconazole, known for its antifungal efficacy, it can be pointed out that in most cases, and as however generally expected, the positive control has a greater activity than the biotransformation products. The extract containing the compound **3c**, derived from the biotransformation of hyocholic acid, evidenced an efficacy comparable to that exerted by the synthetic antifungal (positive control), in particular against *Phoma betae* and *Sclerotinia sclerotiorum*.

Moreover, the extract deriving from the biotransformation of deoxycholic acid containing the compound **2b** has shown a biological activity comparable to that of fluconazole against *Sclerotinia minor*, so that, also in this case the biotransformation process leads to the advantage of obtaining a molecule that can be potentially used in the agricultural sector as a selective tool in a more sustainable strategy for crop plant defence.

## 3. Discussion

Massive use of the synthetic agropharmaceuticals that have characterised treatments in agriculture in the last century has on the one hand determined an exponential increase in agricultural production in the face of an equally high increase in the human population and in food needs, but on the other hand, problems of environmental pollution (accumulation in water and soil; alterations of ecosystems, etc.) and problems related to human and animal health are emerging. All these critical aspects, also linked to the growing sensitivity of public opinion and politics towards a more sustainable management of production, have led to the need for more sustainable and biocompatible crop control tools [23,24,25,26].

This work was focused on the evaluation of the anti-phytopathogenic activity of bovine and porcine bile acids and extracts containing molecules deriving from the biotransformations of the same bile acids, called secosteroids. The targets used for this research were ten phytopathogenic fungi that cause multiple problems affecting the agricultural sector worldwide.

Each pathogen was grown in a medium containing 1000 µg/mL of test substance, a concentration coherent with potential use in the field. In this phase both the secosteroids and the bile acids were tested to be compared. For the diametrical growth inhibition determination, the fungal growth of the treated phytopathogens was compared to that of the negative controls.

Molecules that gave an inhibition percentage greater than-or equal to-80% were tested again in a concentration range of 25–1000 µg/mL, to check the possible dose–response effect and to compute the IC_50_ values.

In general, the data obtained with fluconazole provide the best results but those obtained with *Phoma betae* treated with deoxycholic acid are quite interesting because deoxycholic acid is a surface-active agent (and not a specific antifungal agent). It is a waste product, obtained from the processing of bovine and pig bile to produce ursodeoxycholic acid, a drug used in the treatment of gallstones. Moreover, recently, deoxycholic acid has been approved for use in the cosmetic field in the treatment of imperfections [72] as well as being known to inhibit the growth of *Candida albicans* [73]. This aspect supports the substantial safety of the compounds useful for facilitating the possible path towards their application on a large scale in agriculture, since the regulatory aspects in this context are in any case stringent and different from the already consolidated areas of application. In fact, to the best of our knowledge the application to the agricultural field is completely new for these kinds of compounds and, therefore, it is promising to avoid negative environmental impact, toxicological effects on humans, animals and plants and resistance phenomena to treatments by phytopathogens which, on the other hand, can characterise normal treatments with synthetic pesticides. The only study present reports that deoxycholic acid can induce complementary lines of defence, such as callose deposition, reactive oxygen species accumulation and jasmonic acid and salicylic acid signalling pathways [74].

For the biotransformation products, the positive control in most cases has a greater activity than the biotransformation products. However, it could be hypothesised that even if the inhibition effect occurs at higher concentrations than the control, the potential absence of resistance effects make them anyhow promising as natural alternatives to synthetic compounds. Furthermore, their use cannot be excluded in any case, but instead can be supported-if not as an alternative-at least as a complementary support to treatments with synthetic compounds. In fact, this type of integrated approach would make it possible to reduce the quantity of synthetic compounds used, counterbalanced by the use of natural effective biotransformation compounds, reducing in any case the impact on the environment and all the critical aspects already extensively highlighted [23,24,25,26].

In the literature, there are few studies where microbial biotransformations have been used to obtain new antifungals. However, among the most relevant and related research studies, Bajpai et al. reports that molecules derived from the biotransformation of eicosapentenoic acid and docosahexenoic acid lead to the formation of compounds active on *Botritis cinerea* and *Fusarium oxysporum*, respectively [75,76].

Of particular interest was the extract deriving from the biotransformation of deoxycholic acid containing the compound **2b** as it has shown a biological activity comparable to that of fluconazole against *Sclerotinia minor*, so that, also in this case, the biotransformation process leads to the advantage of obtaining a molecule that can be potentially used in the agricultural sector as a selective tool in a more sustainable strategy for crop plant defence. There are no known biotransformation products active on this fungus, however there are some in vitro studies where extracts containing steroid-derived molecules are used in the fight against this pathogen. Some examples are represented by solamargin and solasonin, steroidal glycoalkaloids isolated from eggplants (*Solanumn melongena*) that showed a protective efficacy against the attacks of fungi, bacteria, and phytophagous insects [77]. The EC_50_ values (15–25 µg/mL) obtained with extracts from eggplant leaves containing these molecules pointed out the capacity against phytopathogens with efficacy comparable with that obtained against *Sclerotinia sclerotiorum* and *Phoma betae* with the extract containing product **3c**.

Results obtained with the extract deriving from the biotransformation of hyocholic acid containing the compound **3c** against *Phoma betae* and *Sclerotinia sclerotiorum* points out an important selectivity in exerting biological activity against phytopathogens, supporting the safety of the biotransformed compound against non-target microorganisms. To date, hyocholic acid is considered a full waste from the processing of porcine bile and no valorisation approach has been attempted so far. The only scientific evidence regarding its biological activity is in the regulation of glucose homeostasis, and therefore in a totally different context from the one highlighted here [78].

To the best of our knowledge, in the literature there are no studies about the antifitopathogenic activity of biotransformed products against *Phoma betae*. On the contrary, interesting results are reported about *Sclerotinia sclerotiorum*, but concerning the antifungal properties of bioconverted products starting from different substrates than those used for this study. In particular, the results referred to anti-fungal properties of several bioconverted extracts obtained starting from hydroxylated fatty acid mixtures employed as substrate, such as ricinoleic and eicosadienoic acids, produced by the biotransforming activity of a *Pseudomonas* PR3 bacterial strain [79]. These extracts showed antifungal activity against *Sclerotinia sclerotiorum* with MIC value of 500 µg/mL, comparable to those obtained with the extracts **2a**, **3c** and **4d + 5d**.

Thanks to the biotransformation process, these products, currently considered wastes, could be recovered and potentially used as an effective tool to control phytopathogens in agriculture, promoting a more sustainable way to manage crop plant defence.

## 4. Materials and Methods

### 4.1. Microrganisms

In this study the following 10 phytopathogenic fungi were used: *Alternaria* sp. (ATCC 20084); *Botrytis cinerea* (ATCC 11542); *Fusarium moniliforme* (ATCC 36541); *Fusarium oxysporum* (ATCC 48112); *Neonectria galligena* (ATCC 11684); *Phoma betae* (ATCC 24797); *Penicillium crustosum* (ATCC 52044); *Phytium ultimum* (CBS 29131); *Sclerotinia minor* (ATCC 52583); *Sclerotinia sclerotiorum* (ATCC 46762).

### 4.2. Secosteroids Synthesis

The tested biotransformation mixture extracts containing secosteroids were obtained as previously reported by Costa et al. [70,71]. The products obtained were the following: 3,7α,12α-trihydroxy-9-oxo-9,10-seco-23,24-dinor-1,3,5(10)-cholatrien-22-oic acid **2a** from biotransformation of cholic acid; 3,12α-dihydroxy-9-oxo-9,10-seco-23,24-dinor-1,3,5(10)-cholatrien-22-oic acid **2b** from biotransformation of deoxycholic acid; 3-hydroxy-6,9-epoxy-9,10-seco-23,24-dinor-1,3,5(10),6,8-cholapentaen-22-oic acid **3c** from biotransformation of hyocholic acid; 3,9β-dihydroxy-6x,9α-epoxy-9,10-seco-23,24-dinor-1,3,5(10)-cholatrien-22-oic acid **4d** and 3,4,9β-trihydroxy-6β,9α-epoxy-9,10-seco-23,24-dinor-1,3,5(10)-cholatrien-22-oic acid **5d** from biotransformation of hyodeoxycholic acid. Products **4d** and **5d** were in the same mixture.

### 4.3. Preliminary Screening

A screening was initially performed to evaluate the biological activity of the compounds against phytopathogenic fungi. In this preliminary phase, Petri dishes (90 mm in diameter) were prepared containing potato dextrose agar (PDA, Difco, Ditroit, MI, USA) integrated first with the starting bile acids, then with the mixtures of biotransformation products at the concentration of 1000 μg/mL. The compounds were dissolved in DMSO at a concentration of 0.1 g/mL, and 0.2 mL of this solution was added to the medium after sterilisation once the temperature of 45 °C was reached [80]. The negative control consisted of PDA medium with the addition of the same concentration of DMSO in the tests (0.1% *v/v*). Subsequently, a piece of mycelium from the stationary phase mother culture was added to each Petri dish [80]. Then the cultures were incubated at 26 ± 1 °C in the dark. The rate of growth inhibition was determined by measuring the mycelium diameter after 7 days and comparing it to the negative control, then carrying out the percentage ratio. All experiments and analyses were repeated in triplicate and averaged.

### 4.4. Mycelial Growth Inhibition

After screening, compounds with fungal activity equal or higher than 80% were identified and these were further investigated. The tests were repeated with the same growth conditions but at different compound concentrations, in particular at 1000; 500; 250; 100; 50 and 25 μg/mL. DMSO (0.1%) was used as negative control and fluconazole as positive control (100 and 50 μg/mL). A disc of mycelium (10 mm in diameter) was used as inoculum and added to each Petri dish. The growth inhibition rate was determined by measuring mycelial diameter (the average of two orthogonal diameters, subtracting the inoculum diameter) daily for 7 days then carrying out the percentage ratio [81,82]. All tests were performed in triplicate and averaged.

### 4.5. Statistical Analysis

Results were expressed as Mean ± SD for the screening and IC_50_ for the mycelial growth inhibition test. Statistical analysis was performed using GraphPad Prism 5 statistical software, by applying mean values using one-way ANOVA to determine if there was a significant difference between the growth of the negative control and that of the compound. A *p*-value of less than 0.05 was considered significant.

## 5. Conclusions

This study investigated the growth inhibition effect induced by bile acids deriving from porcine and bovine bile and the respective products obtained through biotransformation with strains belonging to the *Rhodococcus* genus against some phytopathogenic fungi with a strong impact on many crops of great economic interest in the food supply chain.

Bile acids showed in general low biological activity against phytopathogenic fungi tested. However, deoxycholic acid **1b** instead showed interesting potential as a tool for a selective phytopathogenic fungi biocontrol, since the bioactivity has been selectively expressed against *Phoma betae* with the interesting and significant IC_50_ of 22.14 µg/mL.

On the other hand, considering the derivatives obtained with a biotransformation approach, interesting results have already been obtained from a preliminary screening. The extract containing the secosteroid **3c** towards *Phoma betae* in fact showed an IC_50_ value of 23.95 µg/mL. Similarly, the same extract was found to be effective against *S. sclerotiorum* with a similarly interesting growth inhibition value (IC_50_ of 18.49 µg/mL). Finally, the extract deriving from the biotransformation of deoxycholic acid **2b** has also proved to be a good anti-phytopathogenic agent, in particular towards *Sclerotinia minor* even in comparison to the synthetic compounds (fluconazole). The biotransformation strategy can therefore be a useful process for converting waste molecules into molecules useful for various application contexts, such as that of eco-sustainable treatments in agriculture, explored with this research. The use of bioproducts as tools for a more sustainable management of crop protection is today a necessity to counter the emerging evidence of the environmental and toxicological problems induced up to now by the massive use of synthetic agropharmaceuticals and the phenomena of resistance to treatments, which are more and more widespread.

In conclusion, this study represents the first attempt to valorise such industry by-products through biotechnological approaches for agricultural purposes. The next step of this work involves the design and execution of an applicative study in the controlled environment of greenhouses and in the field (test centre) in order to assess effective potential large-scale applications.

## Figures and Tables

**Table 1:** Most common phytopathogenic fungi in agriculture used as targets in this research.

Fungus	Target Plant Crops	Phytopathy
*Botrytis cinerea*	Pear [38], vine [39,40], citrus [41,42], apricot [43], cherry [44]	Gray rot
*Neonectria galligena*	Pome fruits (apple, pear, quince, medlar) [45,46,47]	Cancer of the pome fruit
*Alternaria* sp.	Asteraceae (lettuce [48], radicchio [49], artichoke [50], sunflower [51]), potato [52], tomato [52], broad bean [53], peas [54]	Black spots, wilt and rot
*Fusarium moniliforme*	Corn [55]	Gray rot
*Fusarium oxysporum f.* sp. *radicis licopersici*	Tomato [56]	Root rot
*Phoma betae*	Chard [57,58]	Foot pain
*Penicillium crustosum*	Cereals [59], apple [60], pear [61], dried fruit [62]	Production of dangerous toxins for animals
*Pythium ultimum*	Cabbage [63], carrot [64], cucumber [65], melon [66]	Root rot and wilt
*Sclerotinia minor*	Soy, sunflower, kidney bean, cucumber, lettuce, spinach, kale, sweet potato, Irish potato, pepper, tomato, peanut [67]	Rust, white mould, rot
*Sclerotinia sclerotiorum*	Herbaceous and succulent plants, especially flowers and vegetables [67,68,69]	White mould

**Table 2:** Antifungal activity screening obtained with bile acids (**1a**–**d**).

Strain	1a	1b	1c	1d
*Botrytis cinerea*	72 ± 3.2%	70 ± 1.7%	78± 1.2%	56± 4.6%
*Neonectria galligena*	52 ± 4.2%	48 ± 2.3%	41 ± 0.4%	15 ± 0.7%
*Alternaria* sp.	66 ± 1.2%	66 ± 2.6%	73 ± 3.3%	66 ± 1.8%
*Fusarium moniliforme*	30 ± 0.5%	83 ± 1.1%	39 ± 0.8%	39 ± 1.7%
*Fusarium oxysporum*	22 ± 2.3%	70 ± 3.5%	48 ± 2.7%	37 ± 2.2%
*Phoma betae*	61 ± 2.5%	100 ± 0%	86 ± 1.8%	87 ± 0.3%
*Penicillium crustosum*	24 ± 4.2%	32 ± 0.7%	8 ± 3.5%	0 ± 0%
*Pythium ultimum*	23 ± 3.5%	78 ± 1.3%	72 ± 0.2%	100 ± 0%
*Sclerotinia minor*	68 ± 1.4%	85 ± 0.7%	56 ± 2.5%	52 ± 3.2%
*Sclerotinia sclerotiorum*	73 ± 4.2%	77 ± 0.9%	80 ± 0.9%	69 ± 3.2%

**1a** = cholic acid, **1b** = deoxycholic acid, **1c** = hyocholic acid, **1d** = hyodeoxycholic acid.

**Table 3:** Antifungal activity screening obtained with secosteroids.

Strain	2a	2b	3c	4d + 5d
*Botrytis cinerea*	30 ± 1.7%	64 ± 3.8%	62 ± 3.9%	78 ± 1.2%
*Neonectria galligena*	36 ± 3.9%	50 ± 4.7%	39 ± 4.2%	56 ± 3.1%
*Alternaria* sp.	0 ± 0%	0 ± 0%	0 ± 0%	0 ± 0%
*Fusarium moniliforme*	34 ± 1.3%	45 ± 2.0%	42 ± 1.9%	95 ± 1.3%
*Fusarium oxysporum*	40 ± 4.8%	40 ± 3.2%	40 ± 3.1%	66 ± 2.9%
*Phoma betae*	37 ± 0.7%	67 ± 0.9%	100 ± 0%	70 ± 1.3%
*Penicillium crustosum*	8 ± 1.7%	12 ± 1.5%	12 ± 2.1%	20 ± 0.8%
*Pythium ultimum*	85 ± 1.2%	55 ± 0.3%	87 ± 0.7%	100 ± 0%
*Sclerotinia minor*	81 ± 0.2%	89 ± 1.1%	93 ± 1.7%	60 ± 0.8%
*Sclerotinia sclerotiorum*	79 ± 0.9%	78 ± 1.1%	100 ± 0%	98 ± 0.3%

**2a** = secosteroid from cholic acid, **2b** = secosteroid from deoxycholic acid, **3c** = secosteroid from hyocholic acid, **4d + 5d** = secosteroids from hyodeoxycholic acid.

**Table 4:** IC_50_ values of bile acids for selected fungi expressed in µg/mL.

Strain	1a	1b	1c	1d
*Fusarium moniliforme*	-	428.9 ± 11.13	-	-
*Phoma betae*	-	22.14 ± 0.98	247.5 ± 6.82	196.6 ± 8.41
*Pythium ultimum*	-	-	-	284.1 ± 4.13
*Sclerotinia minor*	-	377.6 ± 28.75	-	-
*Sclerotinia sclerotiorum*	-	-	712.8 ± 64.22	-

**1a** = cholic acid, **1b** = deoxycholic acid, **1c** = hyocholic acid, **1d** = hyodeoxycholic acid.

**Table 5:** IC_50_ values of secosteroids for selected fungi expressed in µg/mL.

Strain	2a	2b	3c	4d + 5d
*Fusarium moniliforme*	-	-	-	133.3 ± 3.86
*Phoma betae*	-	-	23.95 ± 0.97	-
*Pythium ultimum*	677.0 ± 21.16	-	-	176.2 ± 5.79
*Sclerotinia minor*	730.7 ± 58.26	227.3 ± 19.15	67.90 ± 6.42	-
*Sclerotinia sclerotiorum*	-	135.3 ± 10.28	18.49 ± 1.54	180.98 ± 16.73

**2a** = secosteroid from cholic acid, **2b** = secosteroid from deoxycholic acid, **3c** = secosteroid from hyocholic acid, **4d + 5d** = secosteroids from hyodeoxycholic acid.

## Data Availability

The data presented in this study are available only in the article.
